# *N*-Acetyl Cysteine-Decorated Nitric Oxide-Releasing Interface for Biomedical Applications

**DOI:** 10.1021/acsami.4c02369

**Published:** 2024-05-02

**Authors:** Rashmi Pandey, Vicente Pinon, Mark Garren, Patrick Maffe, Arnab Mondal, Elizabeth J. Brisbois, Hitesh Handa

**Affiliations:** † School of Chemical, Materials, and Biomedical Engineering, College of Engineering, University of Georgia, Athens, Georgia 30602, United States; ‡ Pharmaceutical and Biomedical Science Department, College of Pharmacy, University of Georgia, Athens, Georgia 30602, United States

**Keywords:** antibacterial, antibiofilm, antibiotic resistance, nitric oxide, *N*-acetyl cysteine, polyvinyl chloride

## Abstract

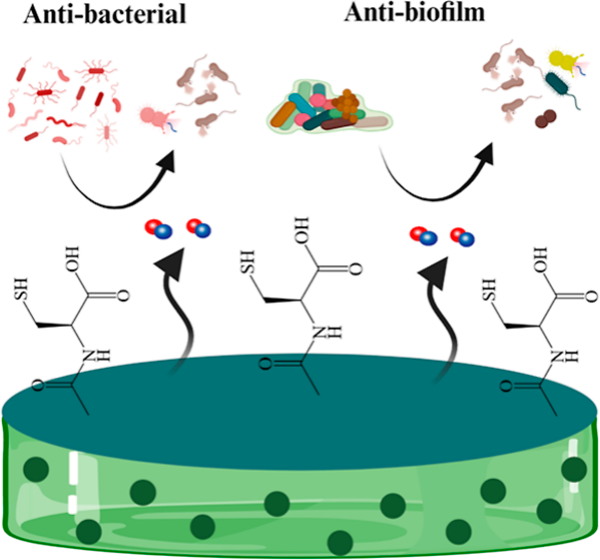

Biomedical devices are vulnerable to infections and biofilm formation, leading to extended hospital stays, high expenditure, and increased mortality. Infections are clinically treated *via* the administration of systemic antibiotics, leading to the development of antibiotic resistance. A multimechanistic strategy is needed to design an effective biomaterial with broad-spectrum antibacterial potential. Recent approaches have investigated the fabrication of innately antimicrobial biomedical device surfaces in the hope of making the antibiotic treatment obsolete. Herein, we report a novel fabrication strategy combining antibacterial nitric oxide (NO) with an antibiofilm agent *N*-acetyl cysteine (NAC) on a polyvinyl chloride surface using polycationic polyethylenimine (PEI) as a linker. The designed biomaterial could release NO for at least 7 days with minimal NO donor leaching under physiological conditions. The proposed surface technology significantly reduced the viability of Gram-negative *Escherichia coli* (>97%) and Gram-positive *Staphylococcus aureus* (>99%) bacteria in both adhered and planktonic forms in a 24 h antibacterial assay. The composites also exhibited a significant reduction in biomass and extra polymeric substance accumulation in a dynamic environment over 72 h. Overall, these results indicate that the proposed combination of the NO donor with mucolytic NAC on a polymer surface efficiently resists microbial adhesion and can be used to prevent device-associated biofilm formation.

## 1 Introduction

Progress within the medical field over the last century has been accelerated by the development of various biomedical devices that contact patients directly or indirectly. Despite being an essential component of healthcare, these devices are at grave risk of infections by opportunistic bacteria. By 2050, bacterial infections associated with antibiotic-resistant strains are estimated to become the number one cause of patient mortality.^1^ The adverse events associated with bacterial infections are exacerbated by the formation of biofilms on the surface. According to the National Institutes of Health, 80% of bacterial infections in humans are associated with biofilm formation.^2^ Free-floating (planktonic) bacteria can attach to any surface, followed by the production of extracellular polymeric substances (EPSs) eventually forming a biofilm.^3,4^ Matured biofilms are well-structured, complex, heterogeneous communities that provide bacteria with structured shelter and help evade antibiotic and host immune system-mediated killing.^3−5^ Upon maturation, bacterial cells embedded in the EPS of the biofilm disperse and migrate to different body parts causing secondary infections including bloodstream infections and sepsis.^4−6^ Current strategies to combat bacterial infections on medical devices include administering antibiotic therapies or removing infected devices.^5^ However, the biofilm EPS layer effectively impedes the diffusion of antibiotics into the matrix, preventing eradication. The high tolerance of antibiotics from these microorganisms has led to the overuse and inevitable development of resistance to these treatments.^3,7^

To counter these limitations, research has been directed toward developing antimicrobial biomaterials that can resist bacterial infections without systemic antibiotics. These strategies include the incorporation of active biocidal molecules, such as silver nanoparticles,^8,9^ antimicrobial peptides,^10,11^ antibiotics,^12,13^ nitric oxide (NO),^11,14,15^ bacteriophages,^16,17^ and surface modifications, such as altered surface wettability^18,19^ and texturing.^20,21^ Despite decades of research, most of these designs have not been commercialized due to a lack of multifunctionality, longevity, or safety concerns. Silver nanoparticle-based coatings have been commercialized but suffer toxicity and reduced efficacy in patients.^22^ Moreover, these coatings cannot resist forming a biofilm for long periods and can eventually get occluded.

Polyvinyl chloride (PVC) is one of the most widely used synthetic thermoplastic polymers for biomedical applications. PVC is nontoxic, inexpensive, stable, sterilization safe, and bioinert, opening it for a wide range of applications, including catheters, blood bags, and medical tubings.^23,24^ However, the hydrophobic nature of PVC allows protein, cellular, and bacterial adhesion limiting its usage.^28,29^

Gasotransmitters such as NO have emerged as a revolutionary approach to fabricate safe, highly antibacterial biomaterials with controlled and localized effects.^25,26^ NO is an endogenously produced free radical gasotransmitter that can regulate various physiological functions such as a platelet inhibitor, antimicrobial, antithrombogenic agent, and wound-healing aid.^25,27,28^ NO can inhibit bacterial growth and trigger the dispersal of biofilms as well as enhance the immune response to infections.^22,29,30^ NO can react with oxygen and oxygen products (such as superoxide and hydrogen peroxide) present in the cells to generate highly toxic reactive oxygen and nitrogen species (ROS and RNS), including hydroxyl radical (OH^–^), peroxynitrite (ONOO^–^), nitrogen dioxide (NO_2_), and dinitrogen tetroxide (N_2_O_3_).^30−33^ These radicals can directly cause cellular damage and bactericidal effects *via* DNA cleavage and mutations (through deamination of bases and strand breakage), protein damage (reacts to thiols and Fe in proteins, causing enzyme inactivation and transcriptional changes), and lipid peroxidation (leading to cell membrane damage).^30,31^ The multimechanistic effects of NO preclude bacteria from acquiring resistance, making it an advantageous candidate as an antibacterial agent.^30,34,35^ The broad-spectrum antibacterial properties of NO have led to its incorporation into polymers to fabricate antibacterial biomaterials which have demonstrated promising results *in vitro* and *in vivo*.^14,15,32,36^ Several NO donors have been developed and incorporated into medical-grade polymers for biomedical applications, such as *S-*nitrosoglutathione (GSNO), *S*-nitroso-*N*-acetylpenicillamine (SNAP), nitroglycerin, and *N*-diazeniumdiolates (NONOates).^37−39^ SNAP has garnered interest as a promising NO donor owing to its ease of synthesis, stability in a polymer matrix, sterilization stability, and biocompatibility.^40,41^ Moreover, incorporating SNAP into a polymer allows for controlled and localized release of NO and can be modulated to achieve long-term release.^40,41^

*N*-Acetyl cysteine (NAC), a safe, inexpensive supplement, has been identified and utilized as a mucolytic agent since the 1960s for chronic lung diseases and emerged as a potent broad-spectrum antimicrobial and antibiofilm agent as both a solution^42,43^ and surface-immobilized moiety.^44−46^ The presence of the free sulfhydryl (–SH) groups in NAC reduces disulfide bonds present in mucus glycoproteins, leading to mucus clearance.^47,48^ This property also bestows NAC with antibiofilm capabilities by interfering in the attachment of EPS proteins, which have been reported extensively against various biofilm-forming bacteria, in both solution and immobilized forms.^45−49^ Although NAC has shown excellent antimicrobial properties against respiratory infections and promising results as a catheter lock solution,^43,45^ only a few implementations of surface immobilization on medical-grade polymers have been previously reported.^44,46^

This study incorporated polyethylenimine (PEI) as a linker molecule to immobilize NAC onto PVC surfaces. PEI is a polycationic molecule with known antimicrobial effects against Gram-positive and Gram-negative bacteria and fungi.^50−53^ PEI, however, has limited applications owing to the high cytotoxic effects on mammalian cells.

This study presents a novel design for biomedical device applications by combining the broad-spectrum antibacterial capabilities of NO with mucolytic NAC and antimicrobial PEI. The surface of PVC blended with a NO donor SNAP is modified using polycationic hyperbranched PEI followed by conjugation of NAC to fabricate a cytocompatible, safe, and innately antibiofilm biomaterial. NO, a broad-spectrum antibacterial agent with multimechanistic antimicrobial capabilities, would reduce microbial adhesion, whereas NAC is expected to prevent biofilm formation on the surface. Moreover, a versatile polymer PVC can be modified to achieve different mechanical and physical properties to cater to specific applications. The PVC–SNAP–PEI–NAC composites made by combining these strategies will enhance the bactericidal and bacteriostatic properties of PVC substrates through decreased bacterial adhesion and enhanced biofilm eradication.

## 2 Material and Methods

### 2.1 Materials

All reagents were used as procured from the vendor unless otherwise mentioned. PVC (high molecular weight), hyperbranched PEI (MW: 25,000), NAC, trioctyl trimellitate (TOTM), tetrahydrofuran (THF), 1-ethyl-3-(3-dimethyl aminopropyl) carbodiimide (EDC), *N*-hydroxysuccinimide (NHS), and Tryptic soy and Luria–Bertani (LB) broth and agar were purchased from Sigma-Aldrich (St. Louis MO, USA). SNAP was purchased from Pharma block (Hatfield, PA, USA). Human fibroblasts [BJ cells, American type culture collection (ATCC) CRL-2522], *Staphylococcus aureus* (ATCC 6538), and *Escherichia coli* (ATCC 25922) were procured from ATCC (Manassas VA, USA). Dulbecco’s modified Eagle’s medium and penicillin–streptomycin were from Thermo Fisher Scientific (Waltham MA, USA). FBS was obtained from VWR (Atlanta, GA USA). All aqueous solutions were prepared in Milli-Q water (18.2 MΩ) and purified using a Mettler Toledo (Columbus, OH USA) apparatus. The drip flow bioreactor apparatus was acquired from BioSurface Technologies Corporation (Model: DFR-110-6, Bozeman, MT, USA).

### 2.2 Fabrication of PEI and NAC-Immobilized NO-Releasing PVC Composites

#### 2.2.1 SNAP-Blended PVC Composite Fabrication

PVC composites with and without the NO donor SNAP were cast by a solvent evaporation method. Briefly, 500 mg of high-molecular-weight PVC was dissolved in 10 mL of anhydrous THF and different concentrations of plasticizer TOTM (1, 2, 3, 4, 5, and 10%) by stirring on a magnetic hot plate. Fifteen milliliters of the polymer solution was poured into Teflon molds (3 × 5 cm) for composite casting using a solvent evaporation technique. Similarly, composites were prepared with SNAP (20 wt % of the total mass). The solvent was allowed to evaporate in a fume hood overnight. The composites were rinsed with DI water and dried under vacuum for another 24 h for complete removal of THF.

#### 2.2.2 PVC–PEI Conjugate Preparation and Dip-Coating

PVC was modified with amines of PEI via a substitution reaction as reported earlier with minor modifications.^54^ Briefly, 500 mg of PEI was reacted with 10 mL of PVC solution (10 mg mL^–1^) in THF at 45 °C for 18 h. The solution was precipitated and washed twice with ice-cold DI water by centrifuging at 2000*g* for 20 min. The rigorous washing was performed to remove any unreacted water-soluble PEI and potential byproducts. The precipitated product was lyophilized to obtain PVC–PEI conjugate. The PVC–PEI conjugate was dissolved in THF at concentrations of 10, 20, 30, 40, and 50 mg mL^–1^ and supplemented with the plasticizer TOTM. Pristine PVC composites were dip-coated twice with the PVC–PEI conjugate dip-coating solution and dried in a fume hood overnight.

#### 2.2.3 NAC Conjugation

NAC was linked to the PVC–PEI-coated composites using EDC–NHS coupling. Briefly, the PVC–PVC–PEI composites were sequentially incubated with EDC (20 mM), NHS (40 mM), and NAC (40 mM) solutions for 1, 2, and 24 h, respectively, at room temperature. The final composites were rinsed with DI water and dried in a desiccator. All the composites were stored at −20 °C and protected from light.

### 2.3 Material Characterization

The fabricated composites were characterized to study the physical and mechanical properties of the novel biomaterial. Overall, the following sample types were prepared and characterized as listed in Table 1.

**Table 1:** List of Samples Fabricated

sample	abbreviation
control PVC	PVC
PVC + PVC–PEI-coated composites	PVC–PEI
PVC + PVC–PEI coated + NAC immobilized	PVC–PEI–NAC
PVC + SNAP blended + PVC top coated	PVC–SNAP
PVC + SNAP + PVC–PEI coated + NAC immobilized	PVC–SNAP–PEI–NAC

#### 2.3.1 Mechanical Properties

The tensile strength of the composites was measured using a Mark-10 ESM303 motorized test stand. Composites were prepared in uniform rectangles (1 cm × 4 cm) to determine the ultimate tensile strength (UTS). Samples were secured to the motorized test stand using Mark-10 G1103 grip attachments and pulled at a speed of 10 in min^–1^. Samples were deemed broken at a 50% break detection. At this point, the max load (*N*) was recorded and normalized to the cross-sectional gauge area of the sample (mm^2^) producing the samples’ UTS value (N mm^–2^).

#### 2.3.2 Fourier Transform Infrared Spectroscopy

Fourier Transform Infrared (FTIR) analysis of the PVC–PEI and PVC–PEI–NAC polymer conjugates was performed using universal attenuated total reflectance sampling of casted polymer composites. Infrared readings were collected from 4000 to 650 cm^–1^ with a resolution of 4 cm^–1^ using a Spectrum 3 FTIR spectrometer from PerkinElmer (Greenville, SC). A total of 128 scans were collected for each sample run, with independently prepared formulations evaluated for each modification.

#### 2.3.3 Water Contact Angle

The surface wettability of the fabricated samples was evaluated by measuring the static water contact angle using an Ossila Contact Angle Goniometer (Model: L2004A1). DI water droplets (10 μL) were placed on the surface, and the water contact angle was recorded and measured using Ossila software. At least three drops of water were placed and recorded on each film, and a minimum of 5 composites were tested for each sample type.

#### 2.3.4 Quantification of Immobilized NAC

The amount of NAC on the film surface was quantified using a colorimetric Ellman’s assay with minor changes to previously published reports.^55,56^ This assay utilizes 5,5′-dithio-bis(2-nitrobenzoic acid) that can react to free sulfhydryl groups to form 2-nitro-5-thiobenzoic acid which has a distinct peak at 412 nm and can be measured spectrophotometrically. Briefly, composites with known dimensions were exposed to 25 μL of Ellman’s reagent (4 mg mL^–1^) and 1.25 mL of reagent buffer and incubated for 15 min at room temperature. The reagent buffer used was 0.1 M phosphate buffer at pH 8.0. The absorbance of the solution was measured at 412 nm using a plate reader (BIOTEK, Synergy, MX, USA). A calibration curve was prepared using cysteine and used to extrapolate the concentration of free thiols, which in turn correspond to the amount of immobilized NAC on the composites.

### 2.4 NO Release and Donor Diffusion

#### 2.4.1 Chemiluminescence Detection of NO Released from the Composites

Real-time evolution of NO from the fabricated composites was measured using a chemiluminescence-based Sievers 280i NO analyzer (GE Analytical Instruments, Boulder, Colorado, USA) as reported in the previous studies.^11,57^ The test composites with known surface area were placed in an amber-colored sample chamber with 3 mL of PBS (10 mM containing 100 μM EDTA) and placed in a water bath at 37 °C. The setup ensures that the NO release is mediated by only a physiological temperature of 37 °C. EDTA is added as a chelating agent to protect NO donors from trace metal ions. A sweep gas (N_2_) was constantly bubbled into the sample chamber at a flow rate of 200 mL min^–1^ that carried the NO released from the samples to the NOA detection chamber. The NO reacts with ozone in the NOA detection chamber and forms nitrogen dioxide in an excited state (NO_2_*). This excited NO_2_ emits a photon while returning to the ground state. A photomultiplier tube amplifies the signal, and the signal is detected by the NOA giving a concentration in parts per billion (ppb). These values were then converted into a NO flux (×10^–10^ mol cm^–2^ min^–1^) using an NOA calibration constant (mol ppb^–1^ s^–1^) and the surface area of the tested composites. The samples were tested over 7 days and were stored at physiological conditions between the readings at 37 °C submerged in PBS.

#### 2.4.2 SNAP Diffusion

To detect SNAP leaching from substrates, preweighed samples were submerged in PBS (10 mM with 100 μM EDTA, pH 7.4) and incubated at 37 °C protected from light. EDTA was added as a chelating agent to prevent the catalysis of SNAP from any trace metal ions present in the solution. The amount of SNAP diffused out in the solution was measured on days 1, 2, 3, 5, and 7 using UV–visible spectroscopy. The absorption of the PBS solution was measured at 340 nm using a UV–vis spectrophotometer (Thermo Scientific). A calibration curve was prepared with known concentrations of SNAP and used for calculating the amount of SNAP in the leachates.

### 2.5 Biological Evaluation

#### 2.5.1 Cytocompatibility Assessment

The cytocompatibility of the fabricated composites was evaluated following the International Organization for Standardization (ISO-10993-5).^58^ An indirect cytotoxicity method was used to test the cytotoxicity of the composites on human fibroblast (BJ) cells. The cells were cultured in tissue culture-treated flasks with appropriate media at 37 °C with 5% CO_2_ in a humidified incubator until confluence and harvested using trypsinization. The cells were seeded on tissue culture-treated 24-well plates (1 × 10^4^ cells per well) and incubated overnight for attachment. The cells were exposed to the composites and placed in sterile hanging cell culture inserts. The total media in each well were 1 mL. The plates were further incubated for 24 and 72 h. After incubation, the media and inserts with composites were removed, and the cells were treated with a CCK-8 reagent and incubated for 1 h at 37 °C. After incubation, the absorbance was measured at 450 nm using a plate reader (BIOTEK). Absorbance at 650 nm was used for plate correction. The Delta OD (Abs_450_ – Abs_650_) was used for analysis. Absorbance of untreated cells was used as control assigning it a 100% viability. The percent viability of the treated samples at different time points was calculated using the following equation.
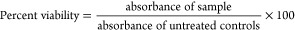


#### 2.5.2 Antimicrobial Efficacy

The antimicrobial efficiency of the fabricated composites was evaluated using two clinically relevant bacterial strains, Gram-positive *S. aureus* (ATCC-6538) and Gram-negative *E. coli* (ATCC-25922) using a previous protocol with minor modifications.^11^ The bacteria were grown in appropriate media until an exponential growth phase and diluted to 10^8^ CFU (colony forming units) mL^–1^ (corresponding to 0.1 OD at 600 nm) in sterile PBS. The bacterial suspension was exposed to surface-sterilized composites for 24 h at 37 °C in a shaker incubator (150 rpm). The adhered bacteria were detached from the composites using homogenization. The adhered and planktonic bacteria were diluted using sterile PBS, plated on agar plates, and incubated at 37 °C overnight to allow bacterial colony formation. Individual colonies were quantified, and total CFU was calculated using the following formula.
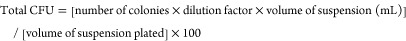


The total CFUs were normalized with surface areas of the composites, and the reduction in bacterial viability was calculated using CFU × cm^–2^ using the following equation.
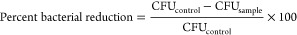


#### 2.5.3 Antibiofilm Efficacy

##### 2.5.3.1 Static Biofilm Assessment

The bacterial cultures were grown as described above and diluted in appropriate media to obtain 10^8^ CFU mL^–1^ (corresponding to 0.1 OD at 600 nm). The bacterial suspension was exposed to sterilized composites and incubated at 37 °C in static conditions to allow biofilm formation for a duration of 72 h with media replenishment every 12 h. After incubation, the composites were removed and gently rinsed with sterile PBS to remove unadhered bacteria. The composites were homogenized to detach the viable adhered bacteria. Samples were diluted and plated on agar plates for quantification. The individual colonies were counted, and the reduction efficiency was calculated as described in the previous section.

##### 2.5.3.2 Dynamic Biofilm Assessment (Drip Flow Bioreactor)

To quantify biofilm formation on substrates in a dynamic environment, a DFR-110–6 Drip Flow Biofilm Reactor (BioSurface Technologies Corporation, Bozeman, MT, USA) was employed to test the extensive antibacterial properties of the material. An overnight culture of *E. coli* ATCC 25922 grown in LB media was washed, resuspended, and diluted to 0.1 OD_600_ (10^8^ CFU mL^–1^). The samples were placed in respective reactor channels and exposed to a 2 g L^–1^ LB media solution containing prepared bacterial suspension for batch-phase biofilm formation. After 8 h, the transition from the batch phase to the drip phase was initiated with a drip flow rate of 0.80 ± 0.03 mL min^–1^ for 72 h at 37 °C and a reactor angle of 10°. After completion of the drip phase, the samples were removed from their respective channels and gently rinsed in PBS to remove unadhered bacterial cells, and the biomass of the biofilm was analyzed via crystal violet (CV) staining at a wavelength of 560 nm to quantify the reduction of biofilm formation on samples. For the Gram-positive dynamic biofilm testing, an overnight culture of *S. aureus* ATCC 6538 was grown in TSB media, washed, resuspended, and diluted to 0.1 OD_600_ (10^8^ CFU mL^–1^). The samples were exposed to a 3 g L^–1^ TSB media solution with the prepared bacterial suspension for batch-phase biofilm formation, and we followed the same procedure as the aforementioned testing with *E. coli* ATCC 25922 for the drip phase and biomass quantification.

##### 2.5.3.3 CV Staining

CV is a cell membrane permeable dye and is used to quantify and visualize biofilm formation. A CV assay was performed following a previous report with slight modifications.^59^ Briefly, samples containing matured biofilms obtained from drip flow bioreactor studies were gently rinsed with PBS and exposed to a 0.4% solution of CV followed by an incubation for 15 min at room temperature. After incubation, the excess stain was removed, and the composites were gently rinsed with DI water. The composites were examined and imaged using a light microscope (EVOS). Another set of biofilms on the film’s surface were stained as described above followed by gentle rinsing. The absorbed CV stain from the biofilms was extracted in acetic acid (30% v/v). The absorbance of the extracted solution was measured at 560 nm using a plate reader (BIOTEK, Synergy, MX, USA).

### 2.6 Statistical Analysis

All the results are represented as average ± standard deviation (SD). All the calculations were performed using Microsoft Excel. Statistical analyses were performed using GraphPad Prism Software (Version 10.0). Ordinary one-way ANOVA coupled with Tukey’s test was employed to calculate the statistical significance. *p* values of <0.05 were considered significant and are reported in the figures. All comparisons were done against plasticized PVC composites as control as well as to each other.

## 3 Results and Discussion

This report presents a novel combinatorial strategy to fabricate a biomaterial by combining the broad-spectrum antimicrobial properties of NO with PEI and NAC. A well-established NO donor, SNAP, was blended into PVC to make NO-releasing composites. These composites were facile-modified via dip-coating with a PVC–PEI conjugate to get an aminated surface on which NAC was immobilized using EDC–NHS coupling. NAC is an antibiofilm molecule used here to resist biofilm formation,^44,49^ whereas PEI is a broad-spectrum antimicrobial agent that can kill bacteria upon contact via membrane permeabilization.^44,50^ NO is a multimechanistic antibacterial^29,60^ agent that also regulates biofilm formation and enhances the susceptibility of bacteria toward antimicrobial agents.^29,61^ Combining these antibacterial mechanisms into one platform resulted in a biomaterial that efficiently reduced bacterial viability and biofilm formation in *S. aureus* and *E. coli* compared to that of individual strategies. The fabricated material was cytocompatible and exerted no significant toxic effects on mammalian cells.

### 3.1 Fabrication and Characterization of PEI and the NAC-Modified Surface

**Figure 1:**
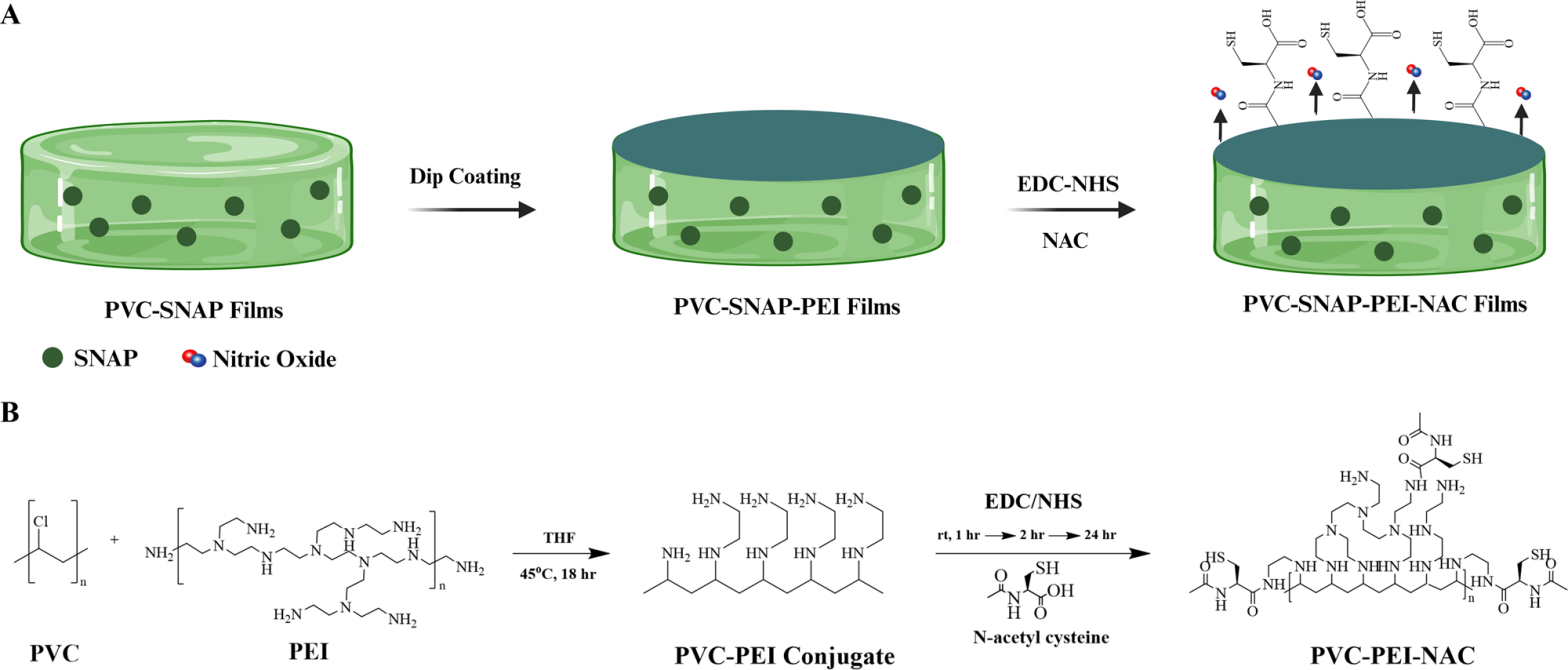
(A) Fabrication of PVC–PEI–NAC composites. SNAP-blended PVC composites were cast using a solvent-casting method. The composites were dip-coated with a PVC–PEI conjugate, and NAC was covalently immobilized using EDC–NHS coupling. (B) Schematic for PVC–SNAP–PEI–NAC composite fabrication. PVC was conjugated with PEI via a substitution reaction. The conjugate when dip-coated on pristine PVC composites provided free amines for further immobilization of NAC onto the surface.

**Figure 2:**
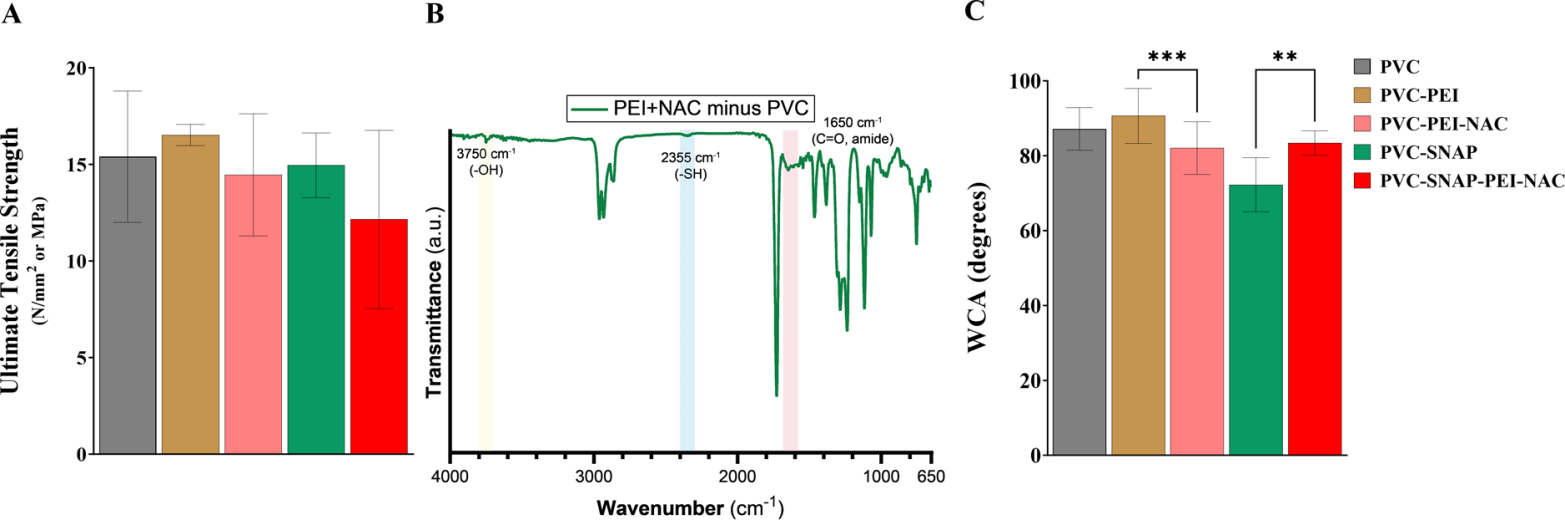
Characterization of the fabricated NO-releasing PVC composites. (A) Tensile testing results of plasticized PVC composites. (B) FTIR analysis of the PVC composites and subsequent modifications. (C) Static water contact angle for PVC composites. Results showing mean ± SD (*n* > 3). ** indicates statistical significance of *p* < 0.01, and *** indicates *p* < 0.001.

The fabrication strategy of the PVC–SNAP–PEI–NAC composites and the schematic for PVC and PEI conjugation and NAC immobilization are detailed in Figure 1. The brittle nature of unplasticized PVC limits its usage in clinical settings. Plasticizers are widely used to modify the mechanical properties of PVC and allow increased malleability, elasticity, and softness required for various applications. TOTM was used as a plasticizer which is a safer alternative to traditionally used phthalate-based plasticizers, which are now recognized as carcinogens.^24,62^ To optimize the plasticizer concentration required, PVC was dissolved in THF supplemented with varying concentrations of TOTM (1, 2, 3, 4, 5, and 10%, volume by volume) and cast into Teflon molds followed by solvent evaporation overnight in a fume hood. The resulting composites were allowed to dry for another 24 h under desiccant to remove any residual solvent completely. The tensile strength of the composites was measured using a Mark-10 ESM303 motorized test stand; the results are summarized in Figure S1. Plasticizer addition with varying concentrations gave a wide range of UTSs between 23.0 ± 3.2 MPa and 1.7 ± 0.2 MPa, allowing the fabrication of composites with desired mechanical properties. Supplementation with 4% volume/volume of TOTM resulted in PVC composites with an UTS of 14.03 ± 3.39 MPa, which was comparable to medical-grade PVC tubing (14.3 MPa, ND 100–65 Tygon, Saint Gobain, PA USA) and was chosen for subsequent characterization. Incorporation of the NO donor SNAP into the polymer matrix via physical blending can alter the mechanical strength of the polymer, as reported by Wo et al.^40^ and Goudie et al.,^41^ with CarboSil and Elast-eon E2A-based materials, respectively. A similar report of SNAP swollen PVC endotracheal tubes demonstrated that SNAP incorporation into PVC does not significantly impact mechanical properties up to a 19.5 wt % age of SNAP.^63^ However, no reports of SNAP blending to bulk PVC have been published. Tensile testing was performed to evaluate the effects of SNAP incorporation via blending and surface modification on the mechanical properties of the composites. The results summarized in Figure 2A indicate that SNAP blending (20 wt %) and the surface modifications slightly decreased the UTS but were not statistically significant. The UTS of SNAP-blended PVC composites was found to be 10.44 ± 4.38 MPa, which was comparable to other reports of SNAP-incorporated PVC tubings.^64^

Next, bulk PVC was modified with hyperbranched PEI via a substitution reaction by stirring at 45 °C for 18 h in THF. The conjugated product was precipitated using chilled DI water and washed twice, followed by lyophilization. The FTIR spectrum of the PVC–PEI conjugates is shown in Figure S2. The synthesized PVC–PEI conjugate was dissolved in THF, and PVC composites with and without SNAP were dip-coated twice with the aforementioned conjugate. The composites were air-dried in the fume hood for 24 h followed by 24 h under vacuum to completely remove THF. The PVC–PEI conjugate dissolved at 20 mg mL^–1^ concentration yielded uniform and smooth dip-coated composites with 5.5 ± 3.2 mM of amines cm^–2^. The amine quantification for various PVC–PEI coatings is shown in Table S1. The composites were further conjugated with NAC using an EDC–NHS coupling method at ambient temperature. The NAC-conjugated composites had 4.1 ± 2.5 mM cm^–2^ of amines on the surface, which was a 73.7% conversion of the surface-available amines. Thiol quantification using an Ellman’s assay demonstrated 356.6 ± 2.7 μM cm^–2^ of NAC on the PVC–PEI–NAC composites. The functional group quantification is summarized in Table S2. Soluble NAC has an antimicrobial activity at a low concentration of 2 μg mL^–1^ against both Gram-positive and Gram-negative bacteria.^65^ This suggests that the significantly higher concentrations of NAC immobilized on the surface should elicit antibacterial effects. The surface modifications were further confirmed using FTIR at every step and are demonstrated in Figures 2B and S3. Characteristic peaks of both PEI and NAC were observed on the final PVC–PEI–NAC conjugate (Figure 2B), including hydroxyl (3750 cm^–1^), sulfhydryl (2355 cm^–1^), and amide (1650 cm^–1^) stretching. These results confirm the successful fabrication of PVC composites with NAC and PEI molecules on the surface. Moreover, covalent immobilization of NAC on the surface is expected to ensure long-term efficacy of the fabricated composites.

The static water contact angle of the fabricated composites was measured to evaluate the surface wettability. Figure 2C shows the contact angles obtained and tabulated in Table S3. The addition of a PVC–PEI conjugate to the PVC surface slightly increased the water contact angle (WCA). The addition of NAC to the surface decreased the WCA significantly compared to that of the PVC–PEI-coated composites (82.8 ± 5.9° compared to 91.8 ± 5.2°, *p* < 0.05). The hydrophilicity induced by NAC is in agreement with a previous report of NAC immobilization.^44^ The blending of SNAP to PVC significantly reduced the WCA compared to that of PVC controls (72.4 ± 6.0° compared to 87.2 ± 5.7°, *p* < 0.0001). Enhanced surface wettability upon SNAP incorporation can be attributed to the formation of hydrogen bonds and has been reported in the literature for other polymeric matrices.^66^ Immobilization of NAC on SNAP-blended composites reverted the water contact angle to 83.4 ± 3.2°, which was not significantly different from NAC-coated composites without SNAP (82.8 ± 5.9°). This phenomenon might be attributed to the addition of slightly hydrophobic PVC–PEI on the surface, effectively encapsulating the SNAP layer and resulting in enhanced hydrophobicity of the composites.

### 3.2 Evaluation of NO Release

**Figure 3:**
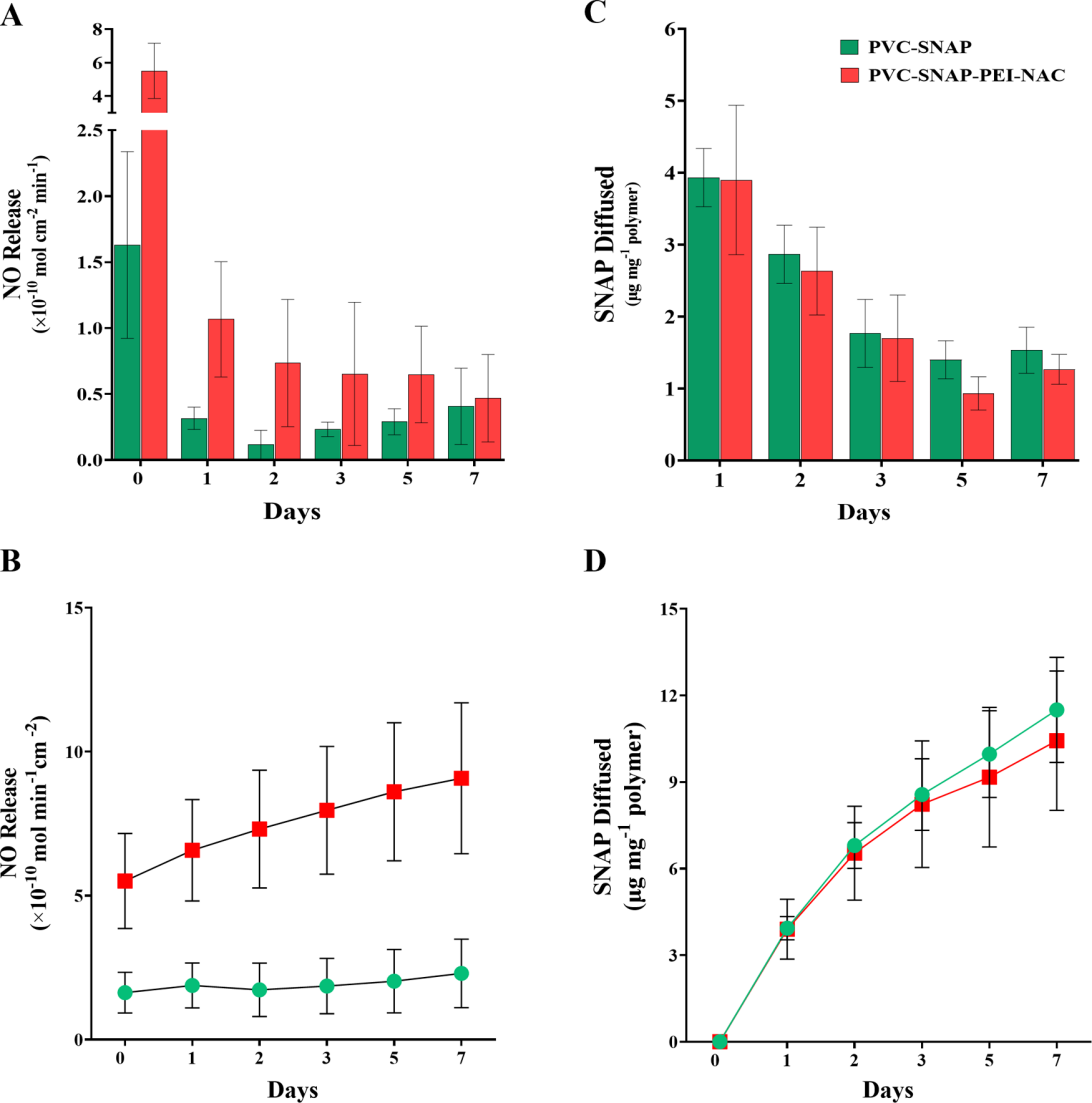
(A) NO release, (B) cumulative NO release, (C) NO donor SNAP diffusion, and (D) cumulative SNAP diffusion from SNAP-blended PVC composites and NAC-immobilized PVC–SNAP–PEI–NAC composites. Studies were done at physiological conditions (37 °C, PBS–EDTA, in the dark) over 7 days. Data represent mean ± SD (*n* = > 3).

NO donor SNAP was incorporated into the polymer matrix via physical blending during film fabrication. The real-time NO release from the fabricated composites was recorded using a chemiluminescence-based NO analyzer under physiological conditions. SNAP can be decomposed and catalyzed by light, heat, metal ions, or enzymes to release NO.^32,39^ The NO release measurements were done in amber vials with a chelating agent (EDTA) to ensure only temperature-induced catalysis of SNAP. Figure 3A summarizes the release of NO from the composites over 7 days. The NO release flux between 0.5 and 4 (×10^–10^ mol cm^–2^ min^–1^) is considered as the physiological levels of NO released from the endothelium.^67^ However, a lower flux of NO up to ∼0.1 to 0.2 (×10^–10^ mol cm^–2^ min^–1^) has shown antibacterial effects in vitro.^68,69^ The PVC–SNAP composites released a 1.63 ± 0.71 (×10^–10^ mol cm^–2^ min^–1^) flux of NO on day 0, whereas the PVC–SNAP–PEI–NAC composites could release a 5.51 ± 1.64 (×10^–10^ mol cm^–2^ min^–1^) flux, which was significantly higher than that of the former (*p* < 0.0001). The NAC-conjugated composites showed a sustained release of NO (>0.5 × 10^–10^ mol cm^–2^ min^–1^) until day 5 and stabilized at a flux of 0.47 ± 0.33 × 10^–10^ mol cm^–2^ min^–^ on day 7 under physiological conditions. The composites evolved NO for at least 7 days with a day-7 flux of 0.41 ± 29 × 10^–10^ mol cm^–2^ min^–1^ for PVC–SNAP composites. The cumulative release of NO from the composites is shown in Figure 3B. The rapid release of a high NO payload on day 0 is beneficial for reducing the initial bacterial viability, which is crucial for infection and biofilm formation on a device surface.^70^ Furthermore, the sustained release of NO can kill any adhered as well as planktonic bacterial cells in the physiological milieu, making the biomaterial resistant to infection for a long period.^69^ These results are indicative of the long-term application potential of the fabricated biomaterial.

Uncontrolled diffusion of the NO donor SNAP into the surroundings can be potentially detrimental for medical applications. Excessive leaching of the NO donor may lead to quick depletion of the SNAP reservoir from the polymer matrix decreasing the longevity of the device application. A previous report has demonstrated the cytocompatibility of SNAP with human fibroblast cells up to a concentration of 0.1 mM (>100 μg mL^–1^).^71^ SNAP diffusion from the PVC–SNAP and PVC–SNAP–PEI–NAC composites was evaluated spectrophotometrically under physiological conditions over 14 days. The SNAP diffusion data are reported in Figure 3B. The PVC–SNAP composites had a cumulative SNAP leaching of 18.5 ± 2.6 μg mg^–1^ of polymer weight, whereas the PVC–SNAP–PEI–NAC composites had 14.2 ± 2.8 μg mg^–1^ of SNAP released over 14 days. These results are comparable with the previously published report of the SNAP-incorporated polymer matrix.^64^ The cumulative release of SNAP from the composites is shown in Figure 3D. The overall concentration of SNAP diffused over 7 days is within the nontoxic concentrations,^71^ illustrating the potential cytocompatibility of the material during extended application durations. The low levels of SNAP diffused from the polymer matrix also indicate that the SNAP reservoir is protected within the matrix and can be used for long-term applications.

### 3.3 Assessment of the Antimicrobial Capabilities of the PVC–PEI–SNAP–NAC Composites

**Figure 4:**
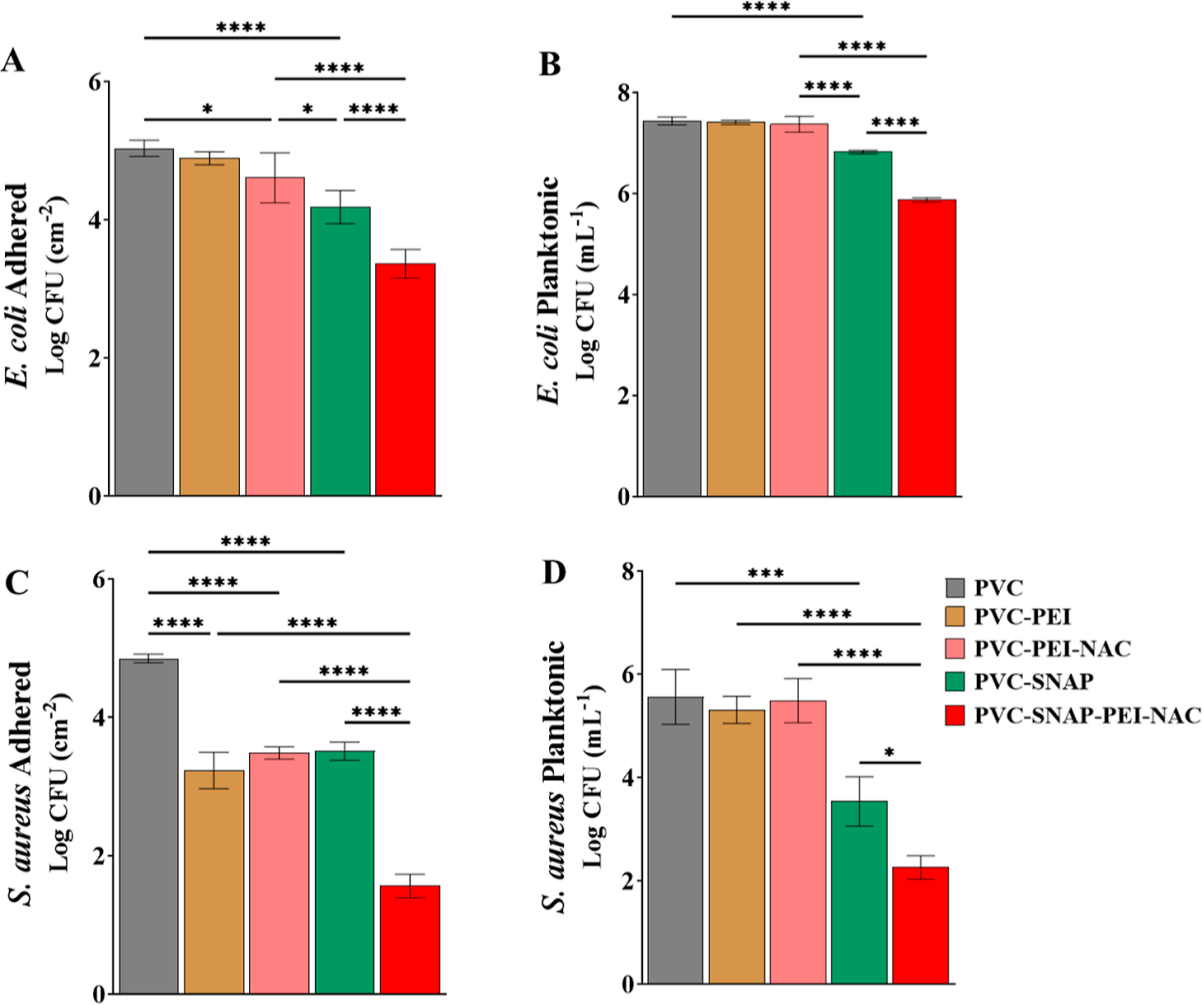
Antibacterial assessment of the fabricated biomaterial showing reduction of (A) adhered *E. coli*, (B) planktonic *E. coli*, (C) adhered *S. aureus*, and (D) planktonic *S. aureus* in a 24 h experiment. Results showing mean ± SD (*n* > 3). * indicates statistical significance of *p* < 0.05, **p < 0.01, ***p < 0.001, and ****p < 0.0001.

A recent report by the Centers for Disease Control and Prevention on adult hospital acquired infections (HAIs) attributed 18% of these infections to *E. coli* and 12% to *S. aureus*, making them the two most notorious culprits for HAIs.^72^ Moreover, it is well-established that bacterial cells can colonize biomedical devices within a few hours of application. Hence, two bacterial strains were chosen to evaluate the antimicrobial capabilities of the fabricated composites over 24 h, followed by an enumeration of adhered and planktonic bacteria. The multifunctional PVC–SNAP–PEI–NAC composites were expected to show superior antibacterial performance over the single strategies. The results summarized in Table 2 and Figure 4 confirm the given hypothesis and demonstrate that PVC–SNAP–PEI–NAC composites to have a significantly lower microbial burden, especially in terms of bacterial adhesion (*p* > 0.05). The PVC–SNAP–PEI–NAC composites reduced viable *E. coli* by 97.74 ± 1.06% and 97.29 ± 0.26% in adhered and planktonic forms, respectively, whereas for *S. aureus*, the reduction was 99.95 ± 0.02% and 99.96 ± 0.01% for adhered and planktonic bacteria, respectively. NO-releasing composites also reduced microbial load significantly compared to that of PVC control. An 84.48 ± 8.57 and 76.11 ± 1.86% reduction in adhered and planktonic *E. coli* and 95.29 ± 1.24 and 91.25 ± 4.19% in adhered and planktonic *S. aureus* was observed with PVC–SNAP composites. These results are comparable with the reported 24 h antibacterial efficacy of SNAP-incorporated polymeric materials with similar NO release profiles for *S. aureus*(64,71) and *E. coli.*(71) Adding the PVC–PEI conjugate on the film surface caused a slight decrease in *E. coli* adhesion (29.60 ± 12.35%) but a significant reduction in *S. aureus* (97.32 ± 1.20%) adhesion. This difference in antibacterial potential against Gram-positive and Gram-negative bacteria has been reported in the literature, where PEI showed selective killing effects against *S. aureus* more than *E. coli*, making Gram-positive bacteria more susceptible to PEI-mediated killing.^51,53^ However, the mechanism for the selectivity is not very clear. Furthermore, NAC conjugation on the surface resulted in a 54.46 ± 27.37% and 95.53 ± 1.81% reduction of adhered *E. coli* and *S. aureus,* respectively. These composites, however, had negligible effects on the planktonic bacterial count with both the bacterial strains tested. NAC is covalently immobilized to the surface and exhibits expected reduction in adhered viable bacteria. The linking prevents the molecule from leaching out to the solution, reducing the killing of free-floating bacteria. This limitation, however, was overcome by combining with the NO release.

**Table 2:** Antibacterial Efficacy of PVC–SNAP–PEI–NAC Composites against Gram-Positive and Gram-Negative Bacteria

sample	change compared to PVC control	E. coli		S. aureus	
		adhered	planktonic	adhered	planktonic
PVC–PEI	log reduction	0.16 ± 0.08	0.03 ± 0.04	1.62 ± 0.22	0.36 ± 0.03
	percent reduction	29.60 ± 12.35	7.20 ± 8.96	97.32 ± 1.20	55.88 ± 3.27
PVC–PEI–NAC	log reduction	0.44 ± 0.36	0.07 ± 0.16	1.41 ± 0.25	0.05 ± 0.19
	percent reduction	54.46 ± 27.37	10.90 ± 34.34	95.53 ± 1.81	1.20 ± 53.11
PVC–SNAP	log reduction	0.86 ± 0.24	0.62 ± 0.03	1.34 ± 0.11	1.10 ± 0.18
	percent reduction	84.48 ± 8.57	76.11 ± 1.86	95.29 ± 1.24	91.25 ± 4.19
PVC–SNAP–PEI–NAC	log reduction	1.69 ± 0.21	1.57 ± 0.04	2.47 ± 1.43	3.37 ± 0.11
	percent reduction	97.7 ± 1.1	97.29 ± 0.26	99.95 ± 0.02	99.96 ± 0.01

Overall, the combination significantly outperformed all the individual strategies in microbial attenuation and potentiated further investigation. The fabricated material has a multimechanistic approach toward bacterial killing and combines the properties of NO, PEI, and NAC onto a single platform. NO can kill a broad spectrum of microbes via ROS and RNS generation, lipid peroxidation, DNA mutation, and protein damage.^30−33^ Moreover, NO being a gas can quickly diffuse into the device’s microenvironment and elicit highly localized and controlled bactericidal effects without exerting systemic consequences. This allows NO to kill planktonic bacteria as well as prevent bacterial adhesion to the surface. Polycationic PEI conjugated to PVC can drive contact killing of bacteria via membrane permeabilization^52^ but showed minimal effects on planktonic bacteria. NAC-immobilized surfaces showed a similar pattern with significant antibacterial efficacy in reducing bacterial adhesion with minimal effects on planktonic bacteria.

### 3.4 Antibiofilm Activity

**Figure 5:**
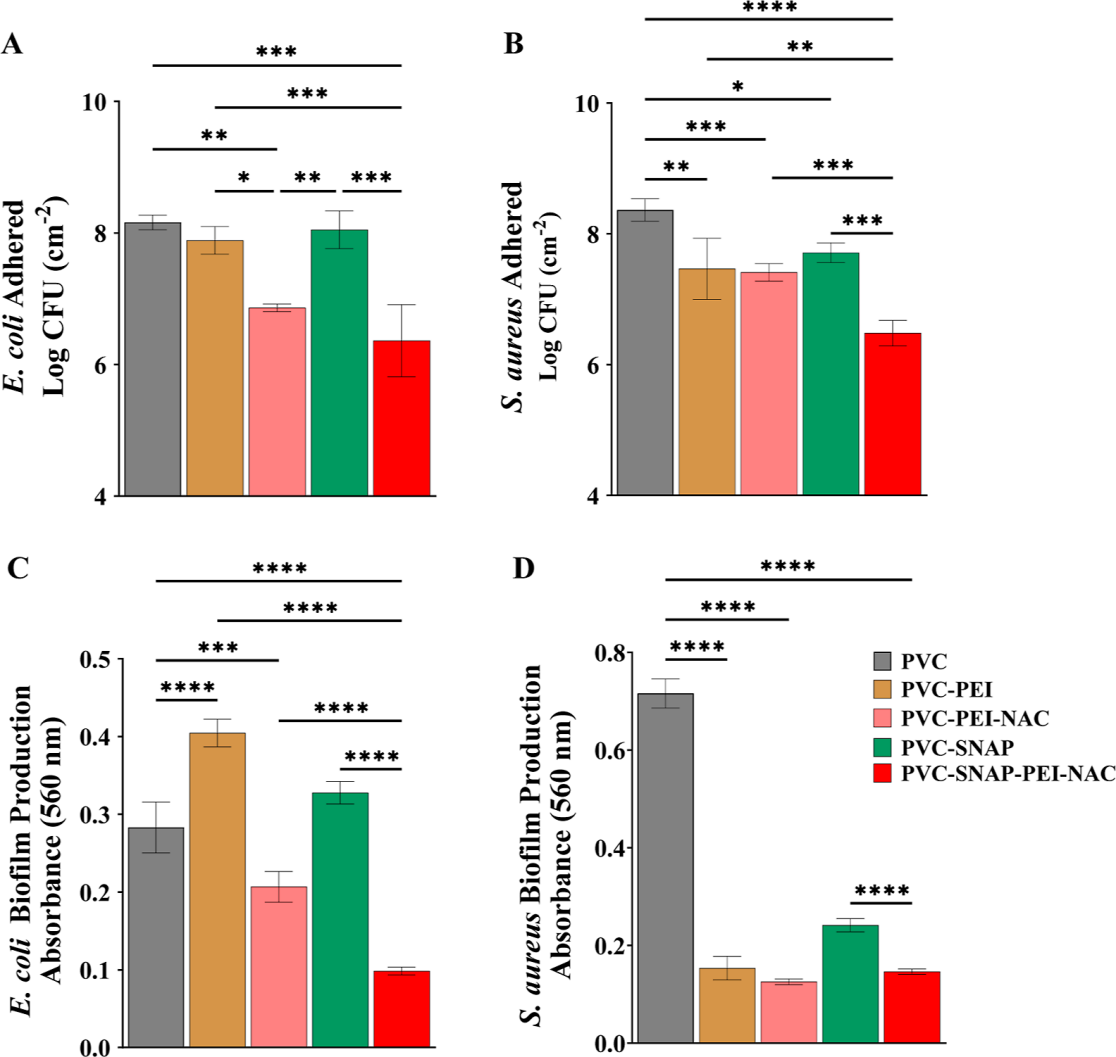
Antibiofilm potential of the biomaterial in a 72 h biofilm formation assay. Viable bacterial counts in biofilms formed under static conditions of (A) *E. coli* and (B) *S. aureus.* CV staining for EPS and biomass estimation using a 72 h drip flow model (C) *E. coli* and (D) *S. aureus.* Results showing mean ± SD (*n* = 3). * indicates statistical significance of *p* < 0.05, **p < 0.01, ***p < 0.001, and ****p < 0.0001.

The promising antibacterial results motivated further evaluation of the biomaterial in preventing biofilm formation on the surface. A biofilm consists of a consortium of microbes embedded inside an EPS that shelters for the microbial colonies to proliferate and evade antimicrobial treatments and immune-mediated killing.^3−6^ The EPS secreted by the microbes consists of various biomolecules such as polysaccharides, proteins, lipids, and water, constituting around 70–90% of biofilm dry mass. The rest of the mass is contributed by microbial cells.^6,73^ Biofilm formation occurs with around 80% of bacterial infections and is a significant contributor to antibiotic resistance development.^9^ The ability of the PVC–SNAP–PEI–NAC composites to prevent the formation of biofilms on the surface was evaluated under static and dynamic conditions. The composites were exposed to 10^8^ CFU mL^–1^ of bacteria and incubated at 37 °C under static conditions to allow biofilm formation. After a 72 h incubation period, the adhered biofilms were detached, and the number of viable bacteria was calculated as described earlier. The results are summarized in Figure 5. The CFU counting results revealed a significant reduction in the number of viable bacterial cells in the biofilm matrix in the PVC–SNAP–PEI–NAC composites. A 97.3 ± 1.9 and 98.7 ± 0.5% reduction was observed compared to that of PVC composites for *E. coli* and *S. aureus*, respectively. PVC–PEI-coated composites reduced the bacterial counts by 43.83 ± 18.63 and 83.6 ± 13.5% for the tested Gram-negative *E. coli* and Gram-positive *S. aureus*, respectively. These observations corroborate the results obtained with initial antibacterial assays, where the PVC–PEI composites showed superior antibacterial efficiency against the Gram-positive strain. NAC-immobilized surfaces further enhanced antibiofilm properties and reduced viable bacterial counts by 95.0 ± 0.7 and 89.1 ± 3.2% for *E. coli* and *S. aureus*, respectively. The antibiofilm properties of NAC-immobilized composites were expected based on previous reports, demonstrating a significant biomass reduction with NAC-modified surfaces.^44,46^ NO-releasing PVC–SNAP composites showed a nonsignificant 14.0 ± 44.0% reduction in *E. coli* counts and a slightly better 78.2 ± 7.3% reduction of *S. aureus* compared to that of PVC controls. These results are similar to that of the antibacterial assays shown in Section 3.3, where SNAP-blended composites performed better against Gram-positive bacteria. These diminished antimicrobial effects can be attributed to the lower NO release from the PVC–SNAP composites compared to that from the PVC–SNAP–PEI–NAC composites. Schoenfisch group showed the dose-dependent effects of NO-releasing PVC-coated xerogels on *Pseudomonas* biofilms.^68^ A lower NO flux (∼1 × 10^–10^ mol cm^–2^ min^–1^) had around an 80% biofilm coverage within 2 h under static conditions, whereas a higher NO flux (∼23 × 10^–10^ mol cm^–2^ min^–1^) showed only about a 20% biofilm coverage. Although past publications have shown potent antibiofilm properties of NO-releasing polymeric substrates,^74,75^ the tested material had significantly higher levels of NO release than that of the PVC–SNAP composites used for this study.

Bacterial cells constitute only ∼10% of a matured biofilm,^75,76^ and a qualitative analysis of bacterial load is insufficient to evaluate the biofilm prevention capabilities of a biomaterial. Additionally, an EPS matrix can allow for easy recolonization even after initial microbial killing.^4^ Hence, for a biomaterial to be truly anti-infective, prevention of EPS formation on the surface is imperative. To assess the capabilities of the biomaterial to prevent formation of an EPS matrix on the surface, a 72 h dri-flow bioreactor was employed. The composites were exposed to 10^8^ CFU mL^–1^ of bacteria and first incubated at 37 °C for 8 h in a batch phase followed by 64 h in a continuous phase. The initial batch phase allowed bacterial adhesion; the next phase mimicked a catheter model with a continuous flow of media. The composites were removed from the bioreactor after 72 h and stained with CV. The results from the CV quantification assay revealed the same efficacy pattern as CFU counts. The results of biofilm prevention studies are shown in Figure 5. Microscopic images of the CV-stained composites are shown in Figure S4. PEI-coated composites did not show any reduction of EPS in *E. coli* but showed a significant reduction for Gram-positive *S. aureus* (78.5 ± 3.3%). These results align with the antibacterial effects of PVC–PEI composites, where a selectivity toward Gram-positive bacteria was observed. Moreover, PEI can cause contact killing of bacteria via membrane permeabilization and polarization but has limited potential against biofilms.^50,76,77^ Addition of NAC to the surface showed slightly better performance than that of PEI with a 27.0 ± 7.0 and 82.5 ± 0.8% reduction for *E. coli* and *S. aureus*, respectively.

The NO-releasing composites had negligible effects on EPS formation by *E. coli* and moderately decreased EPS accumulation for *S. aureus* (66.3 ± 1.9%). These results match the bacterial viability results discussed earlier and can be attributed to the lower flux of NO released from the PVC–SNAP composites. The PVC–SNAP–PEI–NAC composites significantly reduced the EPS content by 65.1 ± 1.8 and 90.7 ± 0.3% for *E. coli* and *S. aureus*, respectively. The reduction obtained with the combinatorial strategy was significantly superior to that with the individual strategies except for *E. coli* CFU reduction by PVC–PEI–NAC composites, which was comparable to the combination. However, the reduction in the EPS matrix for the same sample type was minimal, making it vulnerable to recolonization by bacteria and subsequent infections. Overall, the results taken together demonstrate the superior antibiofilm potential of the PVC–SNAP–PEI–NAC composites to that of the individual strategies and agree with the antibacterial results discussed earlier.

### 3.5 Effects of the PVC–PEI–NAC Composites on Cytocompatibility

**Figure 6:**
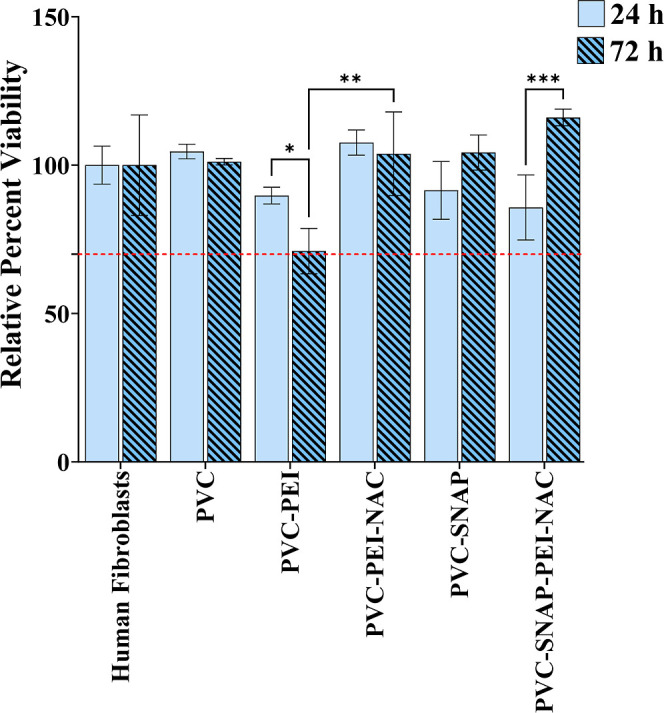
Cytocompatibility evaluation showing percent relative viability of human fibroblast (BJ) cells in a 24 and 72 h indirect toxicity assay. Data represent mean ± SD (*n* > 3). * indicates statistical significance of *p* < 0.05, **p < 0.01, and ***p < 0.001.

Any material exposed to a biological system should possess minimum deleterious effects under physiological conditions. To assess the compatibility of the material to cells, fabricated composites were tested against human fibroblasts (BJ, ATCC CRL-2522) following the ISO standards.^58^ A monolayer of the mammalian cells was exposed to UV-sterilized composites for 24 h at 37 °C with 5% CO_2_ in a humidified incubator using a cell culture insert. The setup allowed any leachates diffusing from the composites to interact directly with the cells for the duration of the experiment. The metabolically active cells were quantified using a tetrazolium dye (CCK-8 reagent), and the percent viability was calculated. ISO guidelines indicate that a reduction of viability by more than 30% is cytotoxic, and hence, the viability of more than 70% compared to untreated cells is desired. The results from the cytocompatibility assay shown in Figure 6 demonstrate that the fabricated PVC composites had acceptable viability against both human fibroblast cells at both 24 and 72 h time points. The incorporation of PEI induced a slight loss in viability at 72 h (71.1 ± 7.6%); however, the values were within the cytocompatible range as per ISO standards. Moreover, the addition of NAC to the surface reverted the slight cytotoxic potential of PEI with a viability of 103.8 ± 14.1% at 72 h. SNAP-incorporated composites had a viability of 91.5 ± 9.7 and 104.2 ± 5.9% at 24 and 72 h, respectively. Polymeric NO-releasing composites have shown proliferative effects on fibroblast cells in vitro,^78^ and similar results were observed in this study. Finally, the SNAP–PEI–NAC composites demonstrated a 85.7 ± 10.9% viability after exposure for 24 h and significantly a higher viability of 116.1 ± 2.8% at 72 h. Overall, the results demonstrate the cytocompatibility, safety, and slight cell proliferative effects of the fabricated composites against model mammalian cells for 72 h.

## 4 Conclusions

This report presents a novel multifunctional biomaterial design strategy combining NO, polycationic hyperbranched PEI, and NAC on a PVC polymer that could efficiently prevent infections while being cytocompatible to mammalian cells. A NO donor molecule, SNAP, was blended into PVC and cast into composites. These composites were facile-modified via dip-coating with a PVC–PEI conjugate to get an aminated surface on which NAC was immobilized using EDC–NHS coupling. The surface modifications were confirmed using FTIR and functional group quantifications. The fabricated composites evolved physiologically relevant NO levels for at least 7 days with minimal leaching of the donor molecule. The antimicrobial evaluation of the material indicated superior microbial killing capabilities of the combination over individual strategies. The composites also effectively resisted biofilm formation on the surface for up to 72 h. Biocompatibility assessment of the composites revealed them to be nontoxic against human fibroblast cells in 24 and 72 h studies. In conclusion, the results from this study show that the proposed design is promising for fabricating anti-infective biomaterials with broad-spectrum antibacterial capabilities. This biomaterial design holds potential for further development after in-depth and long-term evaluation of anti-infective efficacy and biocompatibility.

## Supporting Information Available

Amine quantification of PVC–PEI conjugates; tensile testing of PVC composites with varying concentrations of the plasticizer; functional group quantifications on the composite surface; FTIR of the PVC–PEI conjugate; static water contact angle of the composites; calibration curves; and representative images of CV staining of biofilms (PDF)
