# Supplementary material for: N-Acetyl Cysteine-Decorated Nitric Oxide-Releasing Interface for Biomedical Applications
Source: ACS Appl Mater Interfaces. 2024 May 2;16(19):24248–60. doi: 10.1021/acsami.4c02369 (PMC11103652; doi:10.1021/acsami.4c02369)
Supplement: Supplementary file 1 — am4c02369_si_001.pdf [file am4c02369_si_001.pdf]

Supplementary Information

N-acetylcysteine Decorated Nitric Oxide Releasing interface for Biomedical Applications

Rashmi Pandey, [a] *Vicente Pinon,* [b] *Mark Garren,* [a] *Patrick Maffe,* [a] *Arnab Mondal,* [a]
Elizabeth J. Brisbois, [a] *Hitesh Handa* [a,b] *

[a] School of Chemical, Materials, and Biomedical Engineering, College of Engineering, University of Georgia, Georgia 30602, United States

[b] Pharmaceutical and Biomedical Science Department, College of Pharmacy, University of Georgia, Athens, Georgia 30602, United States

*Corresponding Author:

Dr. Hitesh Handa

Associate Professor

University of Georgia

iSTEM-2, 302 East Campus Rd

Athens, GA 30602

Telephone: (706) 542-8109

E-mail: hhanda@uga.edu

1.0 Amine quantification

The amount of amines present on the surface of the composites was quantified using a Ninhydrin assay developed by Moore [1] with minor modifications. Briefly, the composites were exposed to a 0.2% solution of Ninhydrin reagent and boiled for 10 min at 100 °C. The solution was cooled down to room temperature and the absorbance was measured at 570 nm. The concentration of amines in the samples was calculated using a calibration curve made with glycine.

Amine quantification was also used to determine the PVC-PEI concentration used for the dip-coating. As summarized in **Table S1** the PVC composites dipcoated with 20 mg mL^{-1} of PVC-PEI yielded the most uniform surfaces with minimal variations between different composites. The consistency of the solution was also found to be appropriate for creating a thin coating on the surface without inducing any visible surface roughness. Solutions with higher concentrations had lower solubility leading to an uneven coating and variations in the amine quantification.

Table S1: Amine quantification for PVC-PEI coated composites

**PVC-PEI Concentration** **mg mL^{-1}**	**Amine Concentration** **µM cm^{-2}**
10	51.5 ± 26.4
20	84.3 ± 0.7
30	27.4 ± 11.2
40	25.2 ± 11.7
50	59.1 ± 67.5

2.0 Tensile Testing

Mechanical properties are critical for the functioning of a biomedical device. The tensile strength of plasticized PVC composites was measured using a Mark-10 ESM303 motorized test stand and the data is summarized below. Increasing the TOTM concentration decreased the UTS as expected. The UTS obtained with 4% TOTM was closest to commercially available PVC tubes (14.03 ± 3.39 MPa) and was selected for further studies.

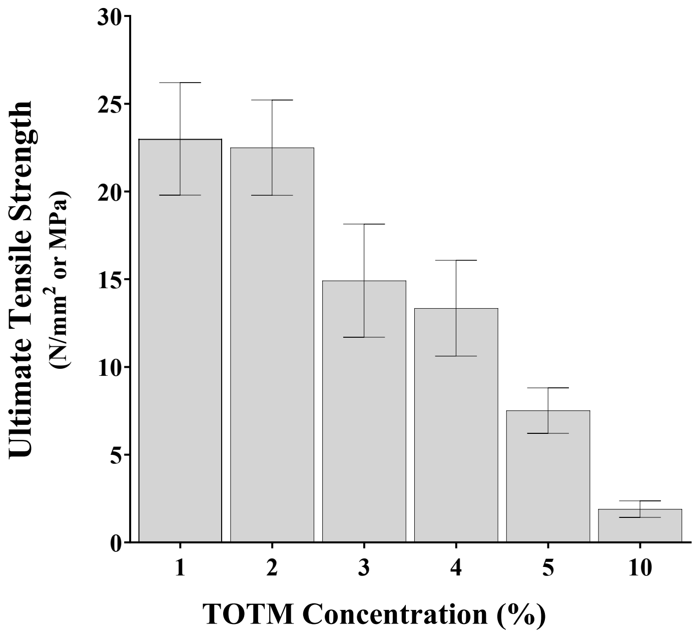

Figure S1: Tensile Testing optimization. PVC (50 mg ml^{-1}) dissolved in THF was supplemented with different concentrations of plasticizer TOTM to cast composites. The tensile strength was measured for each composite using a Mark-10 ESM303 motorized test stand. (n=3 films).

3.0 Functional groups quantification

Table S2: Functional group quantification for PVC-PEI-NAC composites

Amines ($mM\ cm^{-2}$)		Cysteine ($\mu M\ cm^{-2}$)
**PVC-PEI**	**PVC-PEI-NAC**	**PVC-PEI-NAC**
5.5 ± 3.2	4.1 ± 2.5	356.6 ± 2.7

4.0 FTIR

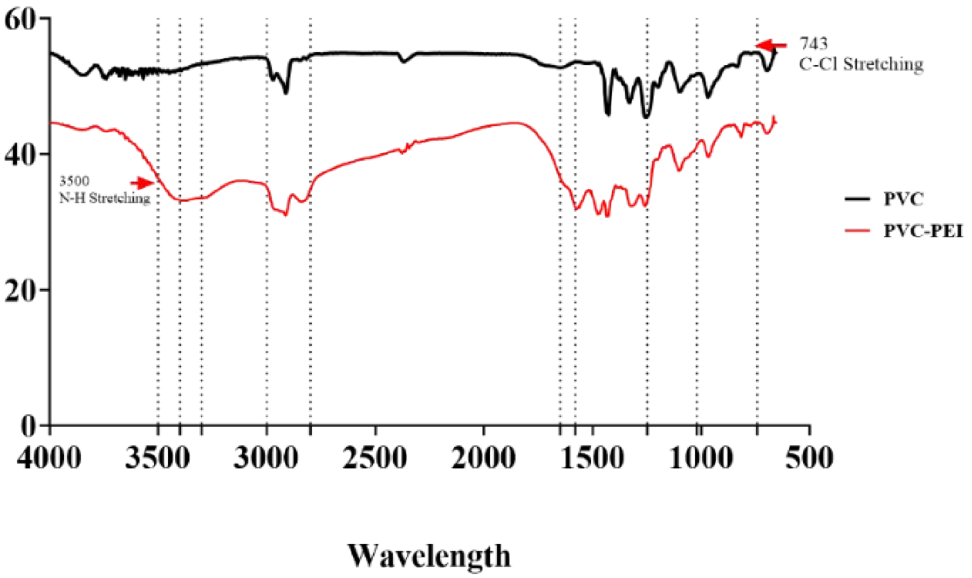

Figure S2: FTIR for PVC-PEI Conjugates indicating the presence of amine groups on the conjugates.

5.0 Water Contact Angle

Table S3: Static Water Contact Angle of the fabricated PVC composites

Sample	Water Contact Angle
PVC	87.2 ± 5.7
PVC-PEI	91.8 ± 5.2
PVC-PEI-NAC	82.8 ± 5.9
PVC-SNAP	72.4 ± 6.0
PVC-SNAP-PEI-NAC	83.4 ± 3.2

6.0 Calibration Curves

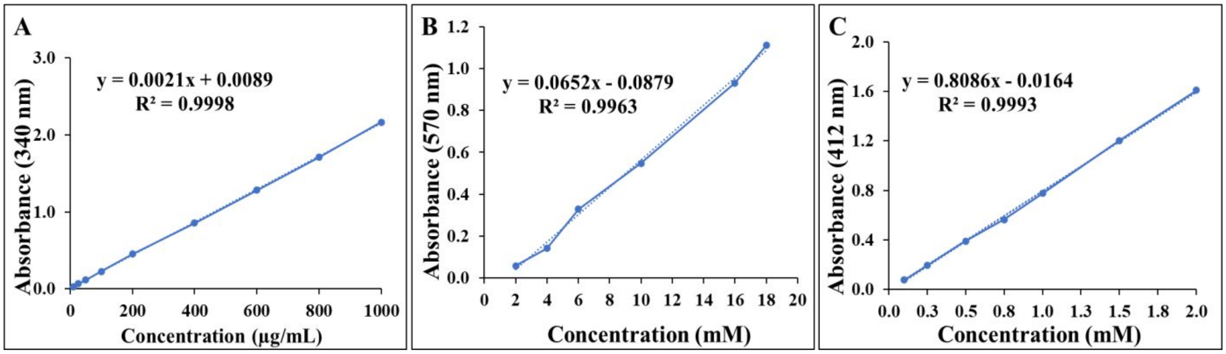

Figure S3: Standard curve of (A) NO donor SNAP in PBS (with EDTA, pH-7.4) used for determining SNAP diffusion at physiological conditions (B) Glycine for ninhydrin assay (amine quantification) (C) cysteine for Ellman's assay (thiol quantification).

7.0 Crystal Violet Staining

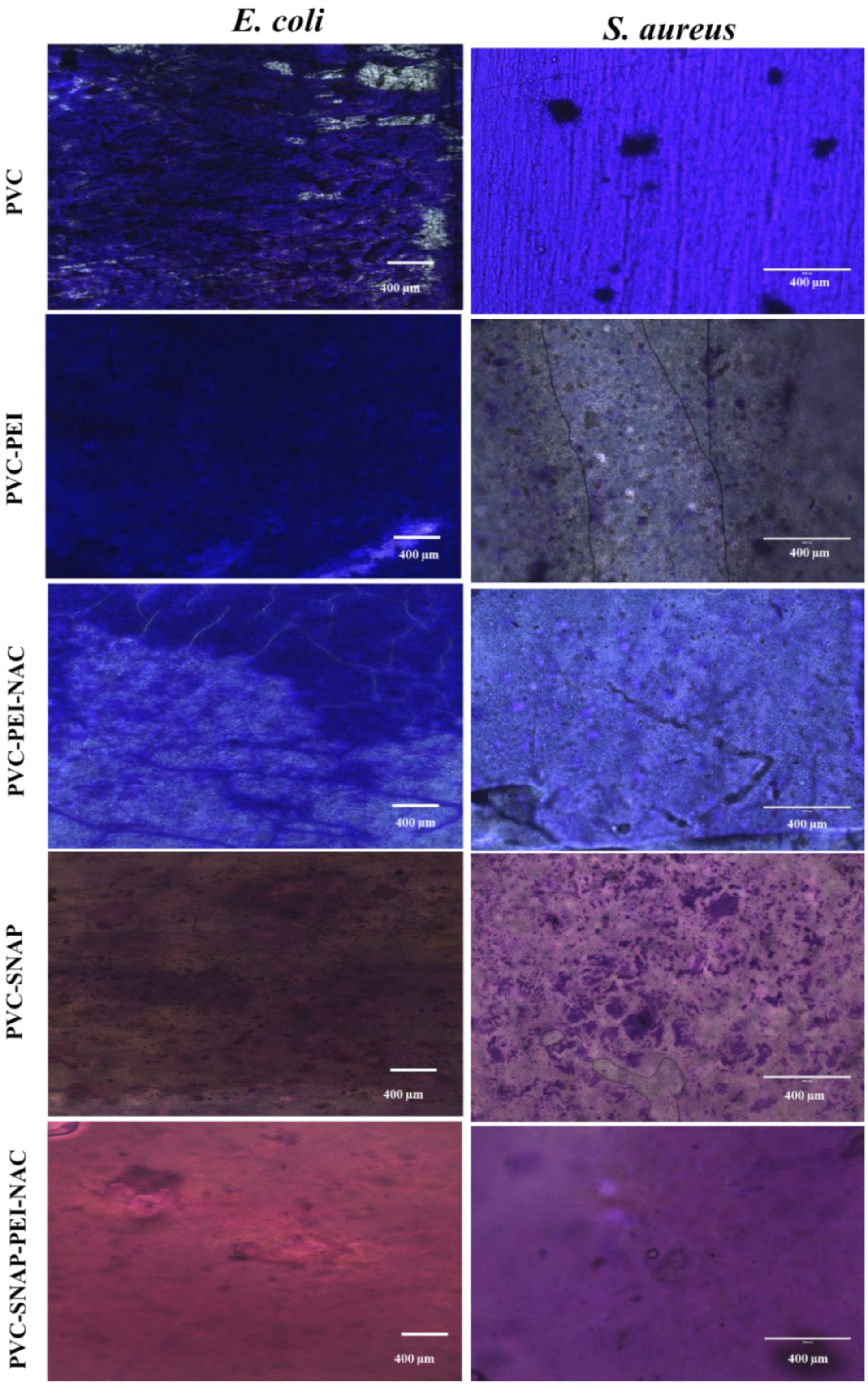

Figure S4: Crystal Violet Staining for visualization of EPS on the material surface. Composites from a 72-h drip flow bioreactor. Scale bar 400 µm.

References:

(1) Moore, S.; Stein, W. H. Photometric nin-hydrin method for use in the ehromatography of amino acids. *Journal of biological chemistry* **1948**, *176*, 367-388.
